# Sickness absence and disability pension after carpal tunnel syndrome diagnosis: A register-based study of patients and matched references in Sweden

**DOI:** 10.1177/14034948211002729

**Published:** 2021-04-12

**Authors:** Tea Lallukka, Rahman Shiri, Kristina Alexanderson, Jenni Ervasti, Ellenor Mittendorfer-Rutz, Marianna Virtanen

**Affiliations:** 1 Department of Clinical Neuroscience, Division of Insurance Medicine, Karolinska Institutet, Sweden; 2 Finnish Institute of Occupational Health, Finland; 3 Department of Public Health, University of Helsinki, Finland; 4 School of Educational Sciences and Psychology, University of Eastern Finland, Finland; 5 Stress Research Institute, Stockholm University, Sweden

**Keywords:** Work disability, occupational factors, entrapment neuropathies, follow-up, population-based

## Abstract

**Aims:** The aim of this study was to examine sickness absence and disability pension (SA/DP) during working lifespan among individuals diagnosed with carpal tunnel syndrome (CTS) and their matched references, accounting for sociodemographic factors. **Methods:** We used a register cohort of 78,040 individuals aged 19–60 years when diagnosed with CTS in secondary health care (hospitals and outpatient specialist health care) and their 390,199 matched references from the general population in 2001–2010. Sociodemographic factors and SA/DP net days during a three-year follow-up were included. Negative binomial regression was used. **Results:** For those not on DP at inclusion, the average number of SA/DP days per person-year was 58 days (95% confidence interval (CI) 56–60 days) among individuals with CTS and 20 days (95% CI 19–21 days) among the matched references. Among both groups, these numbers increased with age and were higher among women than among men. The rate ratio (RR) of SA/DP days was threefold higher among people with CTS than among the matched references (adjusted RR=3.00, 95% CI 2.91–3.10) Moreover, compared to the matched references, the RR for SA/DP was higher among men with CTS (RR=3.86, 95% CI 3.61–4.13) than among women with CTS (RR=2.69, 95% CI 2.59–2.78). The association between CTS and the number of SA/DP days was smaller among older age groups. Sociodemographic factors were similarly associated with SA/DP among people with and without CTS. **Conclusions:** Numbers of SA/DP days were higher among people with CTS than their matched references in all age groups, particularly among individuals in their early work careers, highlighting public-health relevance of the findings.

## Introduction

Musculoskeletal disorders (MSD) and pain are common causes of sickness absence (SA) and disability pension (DP) [1–3]. Previous research has mostly focused on general pain or pain in specific locations, such as back pain and neck/shoulder pain [1–6]. Although carpal tunnel syndrome (CTS) is a common MSD and the most common peripheral nerve entrapment syndrome [7,8], its relations with SA and DP have been addressed in few studies. However, observational studies and clinical trials have assessed time to return to work after carpal tunnel release and found great variation in duration of SA after surgery [9]. Since the symptoms of CTS generally worsen over time, it is important to gain knowledge on predictors and development of SA and DP among people with CTS, in general and in specific age groups. Moreover, although the prevalence of CTS increases with age, it is also relatively common in young adulthood. Therefore, it is important to distinguish between early, mid and late work careers when focusing on consequences of CTS for subsequent work capacity. If CTS similarly leads to higher SA/DP days among younger and older employees, this highlights the need for early detection and intervention. It is also important to explore predictors of SA/DP in people with and without CTS by age in order to identify potential risk groups better.

CTS affects 1–4% of the general adult population, with a clear first notable peak after 50 years of age among both women and men [10–12]. CTS is one of the occupational diseases leading to significant loss of workdays [13], and one of the MSDs with most lost workdays [14]. To date, except for studies on return to work [9], there is limited research on how CTS diagnosed during working life contributes to subsequent labour market participation in terms of SA and permanent exit from paid work in terms of DP. A previous Swedish study on CTS and SA in southern Sweden found that both women and men with CTS had significantly more SA days than their sex-, age- and residence-matched references from the general population [15]. They were followed up for one year before and two years after the diagnosis. However, the study did not consider DP and broader sociodemographic factors, except those used for matching. Thus, for a comprehensive understanding about working days lost after the diagnosis of CTS, both SA and DP days should be considered, including a more comprehensive set of sociodemographic factors, such as indicators of socio-economic position during the life course.

The aim of this study was to examine future SA and DP during working lifespan among people diagnosed with CTS and their matched references, accounting for sociodemographic factors, for all and by age groups.

## Methods

This was a three-year population-based prospective cohort study in Sweden based on nationwide administrative register microdata linked at the individual level.

### Study cohorts

**Figure 1.**
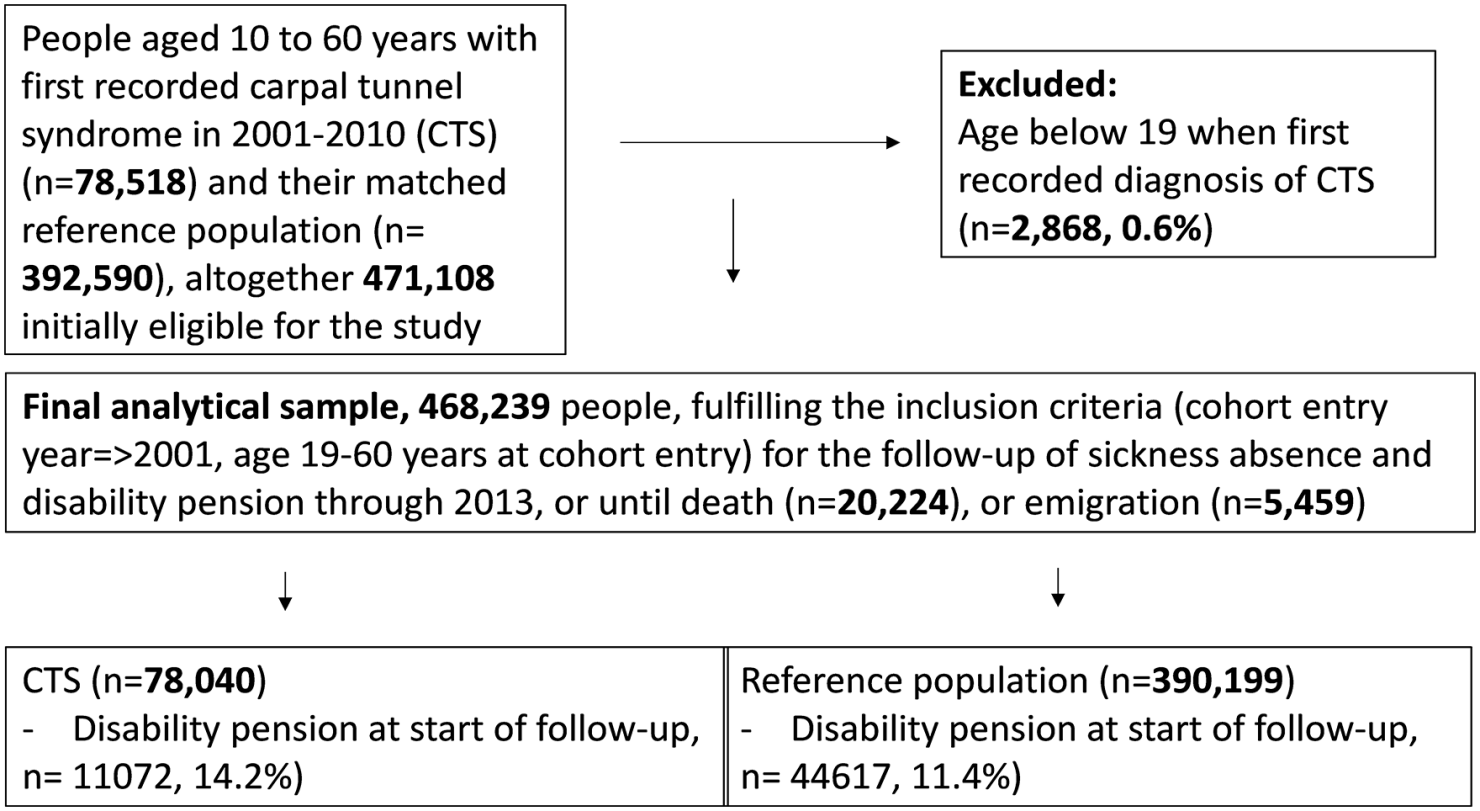
Study population: formation of the data and exclusion and inclusion criteria. CTS: carpal tunnel syndrome.

All individuals with a diagnosis of CTS in secondary health care (hospitals and outpatient specialist health care) in 1995–2010, who were 10–60 years old at the time of their first recorded diagnosis, were included in the initial cohort. As data for outpatient visits were available from 2001 onwards, in this study, we included those first diagnosed in 2001–2010. We included all patients who were diagnosed with CTS at specialised out- or inpatient health care as their main diagnosis (G56.0, ICD-10). The secondary diagnoses were not included. Primary health-care data were not available for this study, but those first diagnosed at primary health care and referred to secondary health care for further investigation, treatment (e.g. carpal tunnel release surgery) or rehabilitation were included in this study. Furthermore, all individuals had to have lived in Sweden for at least five years before their first diagnosis. As CTS is very rare before the age of 19, and as follow-up of SA and DP is meaningful and feasible only after the age of 19, we excluded a small number of those who had their diagnosis before they had turned 19 (0.6%; see Figure 1).

For each person with CTS, we included five matched references from the general population (Figure 1). The randomly selected matched references were to have no indication of CTS in any of the available administrative records before or after the inclusion period and, as among those with CTS, had to have lived in Sweden for at least five years before their cohort entry date (date of diagnosis for their matched CTS individual). We used age, sex, birth country and type of residence area for matching.

Ethical approval for the project was obtained from the Regional Ethical Review Board, Stockholm, Sweden (DNR: 2007/5:6, 2016/1533-32). As this was a fully register-based study, no informed consent was required.

### Register data sources

We used information from several nationwide registers to define people with and without CTS and to derive information on sociodemographic factors, SA and DP. Information from the registers was linked at the individual level using the unique personal identity number assigned to all people registered as living in Sweden [16]. First, we used data from the National Board of Health and Welfare’s patient registers for hospitalisation (from 1964/1987 to 2010) and for specialised outpatient visits (2001–2010) and the death register (date of death). Second, we used the Longitudinal Integrated Databases for Health Insurance and Labour Market Studies (LISA) [17], held by Statistics Sweden, regarding data on living in Sweden, sex, age, country of birth, type of residence area, family situation, parental and own educational level, employment status and occupational group. Third, we used the Microdata for Analyses of Social Insurance (MiDAS), held by the Swedish Social Insurance Agency, regarding SA spells >14 days and DP (start and end dates, and grade (full- or part-time)).

### Sociodemographic factors

Age, sex, birth country, type of residence area, family situation, parental and own educational level, employment status and occupational group were included as sociodemographic factors in December of the year before diagnosed/included (matched references) in the cohort. We categorised all the included individuals into three age groups based on their age at the point of inclusion in order to distinguish between early (19–39 years), mid (40–50 years) and later (51–60 years) work careers. Country of birth was categorised as Sweden, other Nordic countries, EU25 (except Nordic countries) and rest of the world. For the analyses, we dichotomised this to born in Sweden (yes/no). The type of residence area was categorised into three groups: big cities, medium-sized cities and towns/rural areas defined by population size and density. Big cities refer to Stockholm, Gothenburg and Malmö; medium-sized cities are those with more than 90,000 inhabitants within 30 km from the centre of the city and small cities/villages are all the remaining areas of residence. However, in more detailed models, we dichotomised this into big cities versus others. For family situation, we used two categories: (a) married or cohabiting and (b) single (including those who were divorced or widowed). Singles also included a small group of youth (19–20 years) still living with their parents (0.4% of all cohort members). The level of education of the parent with the highest level at the point of inclusion was used. Parental educational level, as an indicator of childhood socio-economic position (life course social position), was categorised into four groups: low (0–9 years; elementary school), intermediate (10–12 years; high school), high level of education (⩾13 years, university/college) and missing information. For about 30% of the individuals, there was no information on parental educational level. This was the case particularly for parents of older cohort members, as such information was not recorded at the time of their education. Participants’ own education was categorised into the corresponding three levels based on their years of education: low (0–9 years and the few missing), intermediate (10–12 years) and high level (⩾13 years). Data on level of education were obtained from Statistics Sweden and initially from the educational authorities in Sweden. The number of years of education at three different levels (elementary, high school and university/college) was used. For immigrants without any education in Sweden, such information is obtained when registering or from directed surveys. Employment status at follow-up start was classified into three groups: (a) employed (including self-employed), (b) not employed and no income from work but recently having been employed (e.g. on leave) and (c) no income from work. Occupational group was dichotomised to distinguish between white- and blue-collar workers. As this information was missing (no occupation) for up to one fifth of the study cohorts, ‘missing’ formed a third category. For that reason, and to avoid over-adjustment, occupational group was analysed only in additional models.

### Sickness absence insurance in Sweden

In the studied years, all people living in Sweden with income from work or unemployment benefits could from the age of 16 be granted SA benefits if their morbidity led to work incapacity. The first day was a waiting day without benefits. For employed, the employer provided benefits for the first 14 days of a SA spell. Thereafter, benefits were provided by the Social Insurance Agency, who also paid benefits for the SA unemployed from day 2. In order not to introduce bias, we included SA spells that had lasted >14 days.

All people aged 19–64 living in Sweden could be granted DP if having long-term or permanent work incapacity due to morbidity. SA benefits amount to 80% of lost income, and DP to about 65%, both up to a certain level. Both SA and DP could be granted for part- or full-time (25%, 50%, 75% or 100%) of ordinary work hours. This means that people could be on partial SA and DP at the same time. Therefore, we calculated the number of net days, also during the first 14 days of the SA spells. For example, two days of absence for 50% equalled one net day. For these reasons, and because SA spells could continue for years, we summed the number of SA and DP days. Ordinary old-age pension age was 65. To produce a comprehensive picture of any SA/DP due to CTS, we included all SA episodes that exceeded 14 days and all DP days without their causes.

### Outcomes

We first modelled crude average numbers of SA/DP net days per person year (days/py) during the three-year follow-up, and then we calculated adjusted rate ratios (RR) for the SA/DP net days over the entire follow-up.

Follow-up for SA/DP days began at the CTS diagnosis date (in 2001–2010) or from the cohort inclusion date for the matched references. Each individual was followed up for three years from this date or until death or emigration, whichever occurred first. During the follow-up, there were 20,224 deaths, while 5459 emigrated. The mean follow-up time was 2.98±0.20 years.

### Statistical analysis

We first computed descriptive statistics for the distributions of sociodemographic factors among people with CTS and their matched references among all and by the three age groups. Second, we estimated the average number of SA/DP net days during the three-year follow-up for all and stratified by sex. Crude number of days/py and adjusted RRs are displayed in the tables. Follow-up time (in years) was used as the offset variable, using a logarithmic transformation. Using the follow-up time as an offset variable enabled us to account for the differences in follow-up times between cohort members, and the logarithmic transformation of the follow-up years enabled the number of SA/DP days/py to be produced. We fitted negative binomial regression models, adjusting for sociodemographic factors and the year of follow-up start (RRs and their 95% confidence intervals (CI)), to compare differences in SA/DP net days between those with and without CTS, and in three age groups reflecting early, mid and later working lifespan. Finally, we examined the associations between sociodemographic factors and SA/DP net days, separately in individuals with and without CTS, and in the three age groups. All RRs displayed were mutually adjusted for all sociodemographic factors, except for occupational group, which was tested separately as described above. For a stronger design, we further repeated each of the above-described analysis excluding all those who were on DP already at baseline. SAS v9.4 (SAS Institute, Cary, NC) was used for the analyses.

## Results

In all, 78,040 patients with CTS and 390,199 matched references were included (Table I). The matching procedure regarding sex, age, type of residence area and birth country was successful (Table I). The mean age of people with CTS and of matched references was 45.1 years. However, there were clear differences in several other sociodemographic factors. For example, people with CTS were more likely to have a lower educational level or to have a blue-collar job. The distributions of study variables were broadly similar for all age groups (Supplemental Table SI). The prevalence of baseline DP was notably lower in the youngest (5.2%) compared to the oldest age group (22.6%) among people with CTS. The corresponding figures among the references were 3.9% and 19.1%, respectively.

**Table I.** Sociodemographic characteristics of individuals diagnosed with CTS when aged 19–60 and their matched reference group without recorded CTS at the beginning of follow-up, among all and among those on DP at inclusion.

Characteristic	All cohort members		Not on disability pension at inclusion	
	People with CTS (*N*=78,040)	References from the general population (*N*=390,199)	People with CTS (*N*=66,968)	References from the general population (*N*=345,582)
*M*_age_ (*SD*)^a^	45.1 (0.04)	45.1 (0.02)	44.2 (0.04)	44.2 (0.04)
Women, %	72.20	72.20	70.75	71.18
Birth country other than Sweden	15.1	15.1	14.30	14.25
Type of residence area				
Large city	28.0	28.0	28.08	28.42
Medium-sized town	35.9	35.9	35.71	35.89
Small town/village	36.1	36.1	36.22	35.69
Family situation				
Married/cohabiting	62.2	60.4	63.23	61.98
Single	37.8	39.6	36.77	38.02
Parental educational level				
High	10.2	14.5	10.92	15.47
Medium	28.5	27.5	29.99	28.83
Low	30.2	27.3	30.25	27.23
Information missing	31.1	30.7	28.84	28.47
Own educational level				
High	21.9	34.2	22.96	36.44
Medium	56.8	49.4	57.45	49.42
Low	21.3	16.4	19.59	14.14
Employment status at follow-up start				
Employed	80.5	80.7	87.62	87.29
Not employed, but with some attachment to labour market	6.5	6.3	5.53	5.86
Not employed	13.0	13.0	6.85	6.86
Occupational group				
White-collar worker	21.9	32.9	23.22	35.64
Blue-collar worker	57.5	45.5	59.57	46.57
Missing	20.7	21.5	17.22	17.78
DP at inclusion	14.2	11.4	0	0

a Age at cohort entry/follow-up start (*SE*).

CTS: carpal tunnel syndrome; DP: disability pension; *SD*: standard deviation; *SE*: standard error.

### All-cause SA and DP during follow-up

Overall, the average number of all-cause SA/DP days/py during the three-year follow-up was 92 days in the CTS cohort and 53 days in the reference cohort (Table II). The number of SA/DP days/py increased with age in both cohorts from 63 days among people aged 19–39 years to 119 days among those aged 51–60 years in the CTS cohort. Corresponding figures were 26 and 81 days/py, respectively, in the reference cohort. The numbers were higher among women than among men in all age groups.

**Table II.** Numbers of SA and DP net days/py and 95% CI in the three-year follow-up among people diagnosed with CTS and their matched references.

	All				Women				Men			
	People with CTS		Matched references		People with CTS		Matched references		People with CTS		Matched references	
	Days/py	95% CI	Days/py	95% CI	Days/py	95% CI	Days/py	95% CI	Days/py	95% CI	Days/py	95% CI
*All*												
Age group (years)												
19–39	63	60–66	26	25–27	68	64–71	29	28–30	48	43–54	18	17–19
40–50	88	84–92	47	46–48	96	91–101	53	52–54	70	64–77	34	33–36
51–60	119	115–124	81	79–82	125	120–130	88	87–90	106	98–115	62	60–64
All	92	90–94	53	53–54	98	95–100	58	57–59	78	74–82	40	39–41
*Excluding those on DP at inclusion*												
Age group (years)												
19–39	50	48–53	15	14–16	53	50–57	17	16–18	40	36–46	9	8–10
40–50	58	56–62	19	18–20	62	58–66	22	21–23	51	46–57	13	12–14
51–60	65	62–69	26	25–26	65	62–69	28	27–29	65	59–72	21	20–22
All	58	56–60	20	19–21	60	58–62	22	21–23	53	50–56	14	14–15

SA: sickness absence; days/py: days per person-year; CI: confidence interval; CTS: carpal tunnel syndrome; DP: disability pension.

Adjusted RRs of SA/DP days/py over the follow-up was higher among people with CTS than among their matched references (RR=2.36, 95% CI 2.30–2.43; Table III). The RR difference between people with and without CTS was larger among men (RR=3.05, 95% CI 2.88–3.22) than among women (RR=2.14, 95% CI 2.08–2.21). Furthermore, among both women and men, RR reduced with age group, and the difference between people with and without CTS in the RR of SA/DP days was larger in younger than in middle-aged or older people. The highest RR was found among men aged 19–39 years (RR=4.12, 95% CI 3.63–4.69).

**Table III.** Adjusted RRs and 95% CI of SA and DP net days during the three-year follow-up among 19-to 60-year-old adults diagnosed with CTS compared to their matched references from the general population without CTS.

	All		Women		Men	
	RR^a^	95% CI	RR^a^	95% CI	RR^a^	95% CI
*All*						
Age group (years)						
19–39	2.92	2.77–3.08	2.60	2.45–2.75	4.12	3.63–4.69
40–50	2.39	2.29–2.50	2.16	2.05–2.27	3.00	2.73–3.31
51–60	1.91	1.84–1.99	1.75	1.67–1.82	2.37	2.18–2.58
All	2.36	2.30–2.43	2.14	2.08–2.21	3.05	2.88–3.22
*Excluding those on DP at inclusion*						
Age group (years)						
19–39	3.35	3.17–3.55	2.93	2.76–3.12	4.74	4.14–5.43
40–50	3.07	2.91–3.24	2.75	2.58–2.93	3.77	3.37–4.21
51–60	2.61	2.47–2.75	2.35	2.21–2.50	3.24	2.91–3.61
All	3.00	2.91–3.10	2.69	2.59–2.78	3.86	3.61–4.13

a Adjusted for age, (sex), birth country, type of residence area, family situation, parental and own educational level, employment status and year when follow-up began.

RR: rate ratio; CTS: carpal tunnel syndrome; SA: sickness absence; DP: disability pension; CI: confidence interval.

### Sample not on DP at inclusion

When excluding those on DP at inclusion (14.2% of people with CTS and 11.4% of people without CTS), the average numbers of future SA/DP days/py were smaller in both the CTS cohort and their matched references than in the full cohort (Table II). However, the difference between people with and without CTS in the future number of SA/SP days during the follow-up was larger. People with CTS had 58 days/py, while the corresponding number for their matched references was 20 days/py (Table II). In all age groups, the number of SA/DP days was higher in women than in men, and the numbers increased with age in both cohorts.

After adjustment for sociodemographic factors, the RR of SA/DP days was higher among people with CTS than among their matched references (RR=3.00, 95% CI 2.91–3.10; Table III). Compared to the matched references, the RR of SA/DP days was higher among men with CTS (RR=3.86, 95% CI 3.61–4.13) than among women with CTS (RR=2.69, 95% CI 2.59–2.78). The strength of the association between CTS and the number of SA/DP days was reduced with age in both women and men.

### Associations between sociodemographic factors and future SA and DP in different age groups among people with and without CTS

All the examined sociodemographic factors were associated with SA/DP among both people with and without CTS (Table IV), but the strength of the associations varied between age groups (Supplemental Table SII). Overall, the RR of SA/DP was highest for non-employment among people both with and without CTS. The RR was also high for those with low educational level (1.66 among people with CTS and 1.92 among people without CTS), but the RRs were also consistently higher for those with a medium level of education (1.45 among people with CTS and 1.55 among people without CTS) compared to those with university/college education.

**Table IV.** Sociodemographic factors associated with the RR (95% CI) of SA and DP among 19- to 60-year-old adults at the cohort inclusion diagnosed with CTS and among their matched references without CTS records.

Characteristics at baseline	Among the total population		Excluding those on DP at inclusion	
	People with CTS (*N*=78,040)	Reference group without CTS (*N*=390,199)	People with CTS (*N*=66,968)	Reference group without CTS (*N*=345,582)
	RR (95% CI)^a^	RR (95% CI)^a^	RR (95% CI)^a^	RR (95% CI)^a^
Sex				
Men	1	1	1	1
Women	1.31 (1.28–1.35)	1.85 (1.80–1.90)	1.23 (1.19–1.27)	1.82 (1.75–1.88)
Birth country				
Sweden	1	1	1	1
Other	1.26 (1.21–1.31)	1.17 (1.13–1.22)	1.38 (1.31–1.44)	1.27 (1.21–1.34)
Type of residence area				
Large city	1	1	1	1
Medium-sized and small towns/villages	1.07 (1.04–1.10)	1.15 (1.12–1.18)	1.08 (1.05–1.12)	1.13 (1.09–1.17)
Family situation				
Married/cohabiting	1	1	1	1
Single	1.16 (1.13–1.19)	1.30 (1.27–1.34)	1.14 (1.11–1.17)	1.27 (1.23–1.31)
Parental educational level,				
High	1	1	1	1
Medium	1.17 (1.11–1.22)	1.11 (1.07–1.16)	1.20 (1.14–1.27)	1.09 (1.03–1.14)
Low	1.15 (1.10–1.20)	1.13 (1.08–1.17)	1.18 (1.12–1.24)	1.13 (1.08–1.19)
Missing^c^	1.17 (1.12–1.24)	1.15 (1.10–1.21)	1.22 (1.15–1.29)	1.17 (1.10–1.24)
Educational level				
High	1	1	1	1
Medium	1.45 (1.40–1.49)	1.55 (1.51–1.60)	1.53 (1.47–1.58)	1.51 (1.45–1.56)
Low	1.66 (1.60–1.72)	1.92 (1.85–1.99)	1.77 (1.69–1.85)	1.77 (1.69–1.86)
Employment status				
Employed	1	1	1	1
Not employed, but with some attachment to labour market	2.06 (1.96–2.17)	2.64 (2.51–2.77)	1.23 (1.15–1.31)	1.22 (1.14–1.30)
Not employed	3.29 (3.17–3.42)	6.86 (6.61–7.11)	1.74 (1.65–1.84)	2.18 (2.05–2.32)
Occupational group^d^				
White-collar worker	1	1	1	1
Blue-collar worker	1.33 (1.28–1.38)	1.39 (1.34–1.44)	1.43 (1.37–1.50)	1.34 (1.28–1.40)
Missing	1.31 (1.25–1.37)	1.43 (1.37–1.49)	1.20 (1.14–1.27)	1.05 (0.99–1.10)

RRs from models mutually adjusting for all variables.

a Adjusted for age, (sex,) birth country, type of residence area, family situation, parental and own educational level, employment status and year when follow-up began.

b Not in the other fully adjusted models, numbers missing are large and could have led to over-adjustment.

Women were more likely to have a higher RR of SA/DP over the follow-up than men (RR=1.85 among people without CTS and 1.31 among people with CTS; Table IV). Birth country, type of residence area, family situation and parental education level were weakly associated with the future number of all-cause SA/DP days. An additional model was fitted including occupational group with all the other variables. Those with a blue-collar job had higher RRs of SA/DP than white-collar workers, irrespective of their CTS status.

## Discussion

This prospective cohort study explored the future numbers of all-cause SA/DP net days after being diagnosed with CTS. Five individually matched references from the general population without CTS for each diagnosed with CTS were included from the same time period. We found that in people who were not on DP at inclusion, the future number of SA/DP days was threefold higher in people with CTS compared to their matched references. A further focus was to examine whether CTS is similarly associated with SA/DP in different work career phases. We found that early in their work career, individuals first diagnosed with CTS – particularly young men – are at the highest risk of future SA/DP. Finally, the associations of sociodemographic factors with future number of SA/DP days were examined. The findings showed that such associations are relatively similar in people with and without CTS in different phases of working lifespan.

### Interpretation

To date, very little is known on future SA/DP in individuals with CTS compared to the general population, particularly including information on DP as well, and sociodemographic factors as predictors in young and older working-aged people. Mostly, studies have estimated time to return to work, which often means the duration of SA. Such results were summarised in a systematic review which highlighted a wide variation in reported return-to-work times after carpal tunnel release surgery [9]. That review also suggested that occupational factors could play a role, it but concluded that in general, they are not well reported and that there is limited evidence available for individual patients regarding the expected length of their SA after surgery. As the focus in the previous studies summarised in the review was on the duration of SA after surgery, the results are not directly comparable to ours. However, despite the different focus, there are similarities in our results compared to the previous ones. For example, our results highlighted that after the CTS diagnosis, SA/DP days were higher among people with CTS than among their matched references. As SA/DP days have not been the primary focus of many trials, it has been emphasised that there is still much to learn not only about the burden of CTS on work capacity, but also the potential of surgery to promote return to work [18]. Even after the publication of the review and further discussion of the need to increase our understanding about return to work after surgery, we only found one recent study showing the lower likelihood of returning to work among blue-collar workers and people with poor preoperative hand function after their surgery [19]. We are not aware of any previous population-based studies focusing on both SA/DP days after the diagnosis of CTS, comparing the net days and RRs of SA/DP days between people with and without CTS, including sociodemographic factors as predictors. Our design using matched references further enabled us to show the additional burden of CTS. Additionally, none of the studies included all recorded CTS, but rather focused on the effects of the surgery only. This study was explorative and aimed to describe comprehensively both the future numbers and RRs of SA/DP days over three years among all people with recorded diagnoses of CTS and their matched references in a nationally representative cohort. More specifically, we included all-cause SA/DP days to produce an overall picture of the occurrence of any SA/DP days. Information on the overall burden of CTS can be used in subsequent studies to focus on, for example, the effects of co-morbidities, specific SA/DP diagnoses, as well as long-term effects of treatments such as surgery on individuals with CTS compared to their matched references. A previous study from southern Sweden of 5456 patients with CTS and their matched references on their SA [15] showed an average number of all-cause SA days per each 30-day period a year before and two years after the CTS diagnosis. The number was higher among people with CTS compared to their matched references. Although the study also had information from primary health care, it did not include DP days in its estimations, nor a wide range of sociodemographic factors, and it did not focus on the risk in different age groups. Thus, the results are only partly comparable, but similarly they show the contributions of CTS to subsequent SA.

Thus, despite CTS being the most common entrapment neuropathy of the upper limbs [7,8], not only its risk factors but particularly its social consequences are not well known. We focused on the social consequences of CTS in terms of future SA/DP days over a period of three years in people aged 19–60 years. The findings indicate that the risk of SA/DP is three times higher in men with CTS and twice as high in women with CTS compared to men and women, respectively, without CTS. The difference between people with and without CTS was even higher among young men and women. As being able to continue in paid work is one of the most important things among people with chronic conditions, this result highlights the need to support work capacity among people with CTS. This is particularly the case among young people during their early careers, and there is a need to implement interventions aiming to maintain, for example, functioning, activity and work capacity and to prevent excess SA/DP days. It is of note, however, that in absolute terms, older women with CTS had the highest number of SA/DP days. Regarding results for those not in work, one could wonder, if the RRs were even higher, if the unemployed did not receive a sick note. However, it needs to be highlighted that in the Swedish system, the unemployed also need a sick note issued by a physician from day 8 of a SA spell. As we only included SA spells that had lasted for at least 15 days, that means that all SA and DP had been diagnosed and assessed as necessary by a physician.

Finally, when interpreting our results, it is important to consider the role of working conditions. On the one hand, exposure to physical workload factors such as lifting or carrying of heavy loads or using vibrating hand tools increases the risk of SA and DP [20–22] as well as the risk of CTS [23]. On the other hand, favourable changes in working conditions can affect the rate of SA/DP [24] as well as the symptoms of CTS. Inclusion of information on occupational group could be assumed to capture to some extent the effects of physical workload on SA/DP, as heavy physical work is more common in the blue-collar group. However, it is of note that working conditions are not considered the leading risk factors for CTS, but rather health-related predictors need to be considered. However, this study lacked information about co-morbidity among those with CTS [25,26] and overall health among their matched references without CTS. It would be an advantage in future studies to include body mass index [27] and other key predictors of CTS.

### Methodological considerations

Main strengths of this study were the large population-based register microdata comprising all registered diagnoses of specialised health care due to CTS in Sweden during the study period, and the recruitment of five references free of CTS for each CTS patient from the general population. The administrative registers used have a very high degree of reliability. The specialised out- and inpatient registers and the LISA register of Statistics Sweden have been shown to be of high quality [16,17,28–30]. We further considered a five-year time period before the start of the follow-up in order to take into account any prior CTS and to exclude those not living in Sweden, that is, without any records to confirm their diagnosis of CTS or SA/DP. Other strengths are the large cohort, allowing for subgroup analyses by sex, age and DP status at inclusion. Except for a rare event of emigration or death, all individuals in the cohort could be followed up for three years (no drop out or attrition, and people who emigrated could be followed up until the date of emigration or death). A further strength is that administrative data were used rather than self-reports, as the latter are affected by recall bias. Also, we included several sociodemographic factors that have been shown to be of importance for SA, DP or both in other studies [31–35]. These factors were used to examine if their associations with SA/DP were similar in individuals with and without CTS. That is of importance, as, for example, blue-collar workers have had longer SA or failed to return to work after carpal tunnel release [9,19].

We did not have information on SA spells shorter than 14 days, which can be seen as both a limitation and a strength. However, the first 14 days of each SA spell were considered in the estimation of net days, as described in the Methods. A limitation is that we had no information about the severity of CTS. In the current study, we included those patients with CTS as their main diagnosis managed within secondary health care. That means that patients who had another hand diagnosis as their main diagnosis and CTS as a secondary diagnosis were not included. This might be the case particularly for middle-aged and elderly patients. Moreover, some patients with CTS seek medical care and receive a diagnosis of CTS in primary health care, but some of these patients with mild to moderate severity do not require further investigation or treatment, and they are not referred to secondary health care. These limitations are important, as it is likely that the rate of SA/DP is smaller in patients with CTS managed in primary health care. The current study may thus have overestimated the overall contribution of CTS to SA/DP days if the number of patients with mild CTS managed exclusively in primary health care was large, as shown previously [36]. However, quite high numbers of working-aged people are diagnosed and treated in outpatient specialist health care and hospitals. However, this should be confirmed in future studies. Another limitation of this study is a relatively short follow-up to establish the long-term contribution of CTS to SA/DP among young employees throughout their working lifespan. However, a longer follow-up could also be problematic, as many other unmeasured factors could emerge and contribute to the SA/DP rates. Another limitation is the lack of data on working conditions, lifestyle factors and other medical conditions.

## Conclusions

This prospective study showed a clear difference between people with CTS and their matched references regarding subsequent SA/DP. The results are emphasised particularly when the focus is on those who were not on DP at the cohort inclusion. Moreover, the risk of SA/DP associated with CTS was particularly high among young people, which highlights the public-health relevance of these findings. In turn, sociodemographic factors appear to have rather similar associations with SA/DP between individuals with or without CTS in all age groups.

## Supplemental Material

sj-docx-1-sjp-10.1177_14034948211002729 – Supplemental material for Sickness absence and disability pension after carpal tunnel syndrome diagnosis: A register-based study of patients and matched references in Sweden Click here for additional data file. Supplemental material, sj-docx-1-sjp-10.1177_14034948211002729 for Sickness absence and disability pension after carpal tunnel syndrome diagnosis: A register-based study of patients and matched references in Sweden by Tea Lallukka, Rahman Shiri, Kristina Alexanderson, Jenni Ervasti, Ellenor Mittendorfer-Rutz and Marianna Virtanen in Scandinavian Journal of Public Health
