# Supplementary material for: Sickness absence and disability pension after carpal tunnel syndrome diagnosis: A register-based study of patients and matched references in Sweden
Source: Scand J Public Health. 2021 Apr 12;50(4):471–81. doi: 10.1177/14034948211002729 (PMC9152599; doi:10.1177/14034948211002729)
Supplement: sj-docx-1-sjp-10.1177_14034948211002729 – Supplemental material for Sickness absence and disability pension after carpal tunnel syndrome diagnosis: A register-based study of patients and matched references in Sweden [file sj-docx-1-sjp-10.1177_14034948211002729.docx]

Supplemental Material

**Supplementary table 1.** Sociodemographic factors of people diagnosed with carpal tunnel syndrome (CTS) when aged 19–60, and their matched references without recorded CTS at the cohort entry in three age groups

| **Characteristic** | **People with CTS (n=78,040)** | | | **References from the general population (n=390,199)** | | |
| --- | --- | --- | --- | --- | --- | --- |
|  | Ages 19–39  (n= 23,440) | Ages 40–50  (n= 26,055) | Ages 51–60  (n= 28,545) | Ages 19–39  (n= 117,200) | Ages 40–50  (n= 130,275) | Ages 51–60  (n= 142,724) |
|  |  |  |  |  |  |  |
| Mean age (SD)^a^ | 32.6 (0.03) | 45.2 (0.02) | 55.3 (0.02) | 32.6 (0.01) | 45.2 (0.01) | 55.3 (0.01) |
| Women % | 75.2 | 69.9 | 71.8 | 75.2 | 69.9 | 71.8 |
| Birth country other than Sweden | 13.0 | 17.8 | 14.5 | 13.0 | 17.8 | 14.5 |
| Type of residence area |  |  |  |  |  |  |
| Large city | 30.0 | 27.7 | 26.7 | 30.0 | 27.7 | 26.7 |
| Medium-sized town | 36.3 | 35.7 | 35.6 | 36.3 | 35.7 | 35.6 |
| Small town/village | 33.6 | 36.6 | 37.7 | 33.6 | 36.6 | 37.7 |
| Family situation |  |  |  |  |  |  |
| Married/cohabitant | 56.1 | 63.2 | 66.2 | 52.7 | 63.8 | 63.6 |
| Single | 43.9 | 36.8 | 33.8 | 47.3 | 36.2 | 36.4 |
| Parental educational level |  |  |  |  |  |  |
| High | 18.7 | 9.0 | 4.4 | 27.1 | 13.0 | 5.4 |
| Medium | 47.5 | 28.8 | 12.7 | 43.6 | 28.9 | 13.1 |
| Low | 23.5 | 35.6 | 30.7 | 18.7 | 32.4 | 29.7 |
| Information missing | 10.4 | 26.6 | 52.3 | 10.6 | 25.7 | 51.7 |
| Own educational level |  |  |  |  |  |  |
| High | 22.2 | 20.8 | 22.6 | 38.6 | 34.1 | 30.8 |
| Medium | 62.3 | 58.8 | 50.6 | 51.8 | 50.8 | 46.2 |
| Low | 15.6 | 20.5 | 26.8 | 9.7 | 15.1 | 23.1 |
| Employment status at follow-up | |  |  |  |  |  |
| Employed | 80.6 | 82.2 | 78.8 | 79.3 | 83.8 | 78.9 |
| Not employed, but with some attachment to labour market | 8.6 | 5.5 | 5.7 | 9.7 | 4.7 | 5.1 |
| Not employed | 10.8 | 12.4 | 15.5 | 11.0 | 11.5 | 16.0 |
| Occupational class |  |  |  |  |  |  |
| White-collar | 19.1 | 21.0 | 24.9 | 30.8 | 33.9 | 33.8 |
| Blue-collar | 60.1 | 59.3 | 53.6 | 47.4 | 46.0 | 43.7 |
| Missing | 20.8 | 19.7 | 21.6 | 21.8 | 20.1 | 22.6 |
| Disability pension at inclusion | 5.2 | 13.1 | 22.6 | 3.9 | 9.8 | 19.1 |

^a^ Age at cohort entry/follow-up start (standard error, SE)

**Supplementary table 2.** Sociodemographic factors associated with the rate ratio (RR; 95% confidence interval, CI) of future sickness absence and disability pension net days among 19-60 year-old adults, diagnosed with CTS, and among their references without CTS records. RRs from models mutually adjusting for all variables in three age groups

|  | Ages 19–39 |  | Ages 40–50 |  | Ages 51–60 |  |
| --- | --- | --- | --- | --- | --- | --- |
| Characteristics at baseline | People with CTS (n=23,440) | Reference group without CTS (n=117,200) | People with CTS (n=26,055) | Reference group without CTS (n=130,275) | People with CTS (n=28,545) | Reference group without CTS (n=142,724) |
|  | RR (95% CI)^a^ | RR (95% CI)^a^ | RR (95% CI)^a^ | RR (95% CI)^a^ | RR (95% CI)^a^ | RR (95% CI)^a^ |
| Sex, women vs. men | 1.45 (1.37–1.53) | 2.20 (2.07–2.34) | 1.39 (1.33–1.46) | 1.93 (1.84–2.02) | 1.17 (1.12–1.23) | 1.58 (1.52–1.64) |
| Birth country, other vs. Sweden | 1.09 (0.99–1.20) | 1.02 (0.91–1.13) | 1.34 (1.25–1.44) | 1.24 (1.15–1.33) | 1.27 (1.19–1.34) | 1.22 (1.16–1.29) |
| Type of residence area |  |  |  |  |  |  |
| Medium-sized and small towns/ villages vs. large city | 1.13 (1.07–1.19) | 1.16 (1.09–1.23) | 1.05 (1.00–1.10) | 1.16 (1.10–1.22) | 1.06 (1.01–1.10) | 1.15 (1.11–1.20) |
| Family situation |  |  |  |  |  |  |
| Single vs. Married/cohabitant^b^ | 1.17 (1.11–1.22) | 1.37 (1.30–1.45) | 1.21 (1.16–1.27) | 1.38 (1.31–1.44) | 1.13 (1.08–1.17) | 1.25 (1.20–1.29) |
| Parental educational level (ref: high) | |  |  |  |  |  |
| Medium | 1.20 (1.11–1.30) | 1.15 (1.06–1.25) | 1.06 (0.98–1.16) | 1.06 (0.99–1.15) | 1.17 (1.06–1.29) | 1.09 (1.00–1.18) |
| Low | 1.15 (1.08–1.23) | 1.16 (1.09–1.24) | 1.07 (0.98–1.16) | 1.05 (0.97–1.13) | 1.11 (0.99–1.23) | 1.06 (0.97–1.16) |
| Missing | 1.27 (1.12–1.43) | 1.09 (0.96–1.24) | 1.07 (0.98–1.17) | 1.14 (1.04–1.24) | 1.17 (1.06–1.29) | 1.15 (1.06–1.24) |
| Educational level (ref: high) |  |  |  |  |  |  |
| Medium | 1.66 (1.56–1.76) | 1.68 (1.58–1.77) | 1.48 (1.40–1.56) | 1.60 (1.52–1.68) | 1.28 (1.22–1.35) | 1.39 (1.34–1.45) |
| Low | 2.20 (2.03–2.39) | 2.73 (2.48–3.00) | 1.69 (1.58–1.81) | 1.99 (1.85–2.13) | 1.39 (1.31–1.47) | 1.56 (1.48–1.64) |
| Employment status at follow-up start | |  |  |  |  |  |
| Employed | 1 | 1 | 1 | 1 | 1 | 1 |
| Not employed, but with some attachment to labour market | 1.48 (1.36–1.61) | 1.59 (1.45–1.74) | 2.34 (2.13–2.56) | 3.75 (3.46–4.06) | 2.51 (2.31–2.73) | 1.65 (1.51–1.81) |
| Not employed | 2.81 (2.61–3.04) | 6.27 (5.76–6.83) | 3.36 (3.14–3.58) | 6.61 (6.30–6.95) | 3.54 (3.35–3.74) | 6.71 (6.17–7.30) |
| Occupational group (ref White-collar)^d^ | |  |  |  |  |  |
| Blue-collar | 1.40 (1.30–1.51) | 1.25 (1.16–1.34) | 1.30 (1.22–1.39) | 1.48 (1.40–1.57) | 1.33 (1.25–1.41) | 1.51 (1.44–1.59) |
| Missing | 1.32 (1.20–1.45) | 1.44 (1.32–1.58) | 1.29 (1.19–1.40) | 1.46 (1.35–1.58) | 1.35 (1.26–1.45) | 1.48 (1.39–1.57) |

^a^ Adjusted for age, (sex,) birth country, residence area, family situation, parental and own educational level, employment status, and year when follow-up began

^b^ not in the other fully adjusted models, numbers missing are large, and could be an overadjustment
